# Kenyan Neonatal Mortality Risk Predictor: Protocol for a User-Centered Design Evaluation

**DOI:** 10.2196/81996

**Published:** 2026-03-27

**Authors:** Ronald Danny Nyatuka, Paul Macharia, Kakhata Esther, Faith Siva, Betsy Muriithi, Md Shafiqur Rahman Jabin

**Affiliations:** 1 School of Computing and Engineering Sciences, Strathmore University, Nairobi, Nairobi, Kenya; 2 Department of Medicine and Optometry, Linnaeus University, Universitetsplatsen 1, Växjö, 39231, Sweden, 46 7915673612; 3 Faculty of Health Studies, University of Bradford, Bradford, England, United Kingdom

**Keywords:** neonatal mortality, machine learning, low- and middle-income countries, feasibility, predictive accuracy

## Abstract

**Background:**

Neonatal mortality remains a major public health challenge in low- and middle-income countries (LMICs), particularly in sub-Saharan Africa, where health systems often lack effective triage mechanisms to identify and prioritize high-risk neonates. Existing clinical tools frequently fail to support timely decision-making during the critical early postnatal period. A previous machine learning (ML)–based neonatal risk prediction model developed using multicountry LMIC datasets demonstrated high predictive accuracy for neonatal mortality in the Indian context, achieving an area under the curve above 0.80. The model incorporates 11 neonatal parameters assessed from delivery through day 2 of life, with birth weight identified as the strongest predictor.

**Objective:**

This study aims to evaluate the contextual feasibility, applicability, and usability of the neonatal risk predictor variables embedded in the ML model in Kenyan health care facilities to inform potential adoption aligned with national health priorities and global targets, including Sustainable Development Goal 3.2.

**Methods:**

A mixed methods feasibility study will be conducted through the real-world preimplementation of a neonatal risk assessment tool in 3 Kenyan health facilities that provide neonatal services. Qualitative and quantitative approaches will be combined to strengthen methodological rigor and enhance the credibility of the findings. The study will be implemented in 3 sequential phases. Phase 1 will involve key informant interviews to capture contextual insights on existing workflows, stakeholder perceptions, and implementation considerations. Phase 2 will comprise a 4-month intervention period during which a paper-based neonatal risk tool will be integrated into routine clinical workflows to assess feasibility, applicability, and usability. Phase 3 will involve postintervention surveys conducted longitudinally at 2 time points to evaluate outcomes, user experience, and implementation barriers.

**Results:**

The neonatal risk predictors are expected to demonstrate strong contextual feasibility, allowing for integration into existing triage systems with minimal workflow disruption. Their applicability is expected to enable early identification of high-risk neonates within the first 48 hours of life, thereby enabling timely clinical decision-making. Usability assessments are expected to indicate positive user experience, acceptability, and perceived usefulness among health care workers. Six participants (frontline neonatal health workers) had been recruited by the time of initial submission of the manuscript (August 2025). Data analysis is ongoing and is expected to be concluded in March 2026. Publication of study findings is expected by June 2026.

**Conclusions:**

The study is expected to generate actionable evidence to support the translation of ML-based neonatal risk prediction into routine clinical practice in LMIC settings, thereby reducing neonatal mortality and advancing progress toward Sustainable Development Goal target 3.2.

## Introduction

### Overview

#### Global Neonatal Health Situation

In 2022, approximately 2.3 million newborns died within the first 28 days of life, averaging 6500 deaths per day and representing 47% of all deaths under the age of 5 years worldwide [1]. Neonatal mortality remains a pressing global health concern, particularly in low- and middle-income countries (LMICs) with limited access to quality maternal and newborn care. Sub-Saharan Africa (SSA) and South and Central Asia bear the highest burden, with SSA recording 27 deaths per 1000 live births and South and Central Asia recording 21 deaths per 1000 live births [1]. Although neonatal deaths declined from 5 million in 1990 to 2.3 million in 2022, progress toward Sustainable Development Goal (SDG) target 3.2, which aims to reduce neonatal mortality to ≤12 per 1000 live births by 2030, remains slow and uneven. Newborns in the highest-mortality countries face a 60-fold higher risk of dying within 28 days than those in high-income countries [1]. These disparities reflect systemic inequities in health systems, socioeconomic conditions, and access to essential care, emphasizing the need to strengthen maternal and neonatal health services in high-burden LMICs.

#### Neonatal Health in LMIC Settings

Neonatal mortality in LMICs is largely driven by preventable conditions such as diarrhea, infections, and respiratory complications [2]. Over 30% of neonatal deaths occur within 24 hours of hospital admission, often due to inadequate triage systems, late presentation, insufficient medical equipment, and personnel shortages [2,3]. Risk factors such as prematurity, respiratory distress, infections, and congenital anomalies exacerbate these challenges. Timely identification of high-risk neonates is critical for rapid emergency interventions to avert preventable deaths. Global neonatal care guidelines advocate for rapid triage, evidence-based treatment, and continuous monitoring; however, implementation in LMICs remains limited [4]. Overcrowded emergency systems; long travel distances; extended waiting times; and shortages of specialists, including neonatologists and pediatricians, further impede effective care [5,6]. These gaps underscore the urgent need for scalable, data-driven tools to support early identification and prioritization of high-risk neonates.

#### Neonatal Health in Kenya

In Kenya, the neonatal mortality rate (NMR) stands at 22 per 1000 live births, reflecting limited access to quality intrapartum and postnatal care [7,8]. Leading causes of neonatal deaths include intrapartum complications; preterm birth; infections; and low birth weight, which affects 8% of Kenyan newborns [9-11]. Infants with low birth weight face mortality rates up to 20 times higher than their counterparts in high-income countries. Over 60% of facilities under the Kenyan Ministry of Health (MOH) lack standardized resuscitation protocols, and staff shortages further compromise timely care. Neonatal triage often relies on subjective clinical judgment, delaying recognition of high-risk neonates [12]. Similar challenges are reported in Uganda and India [13,14]. Addressing these gaps requires automated, evidence-based decision support systems to improve neonatal outcomes.

LMIC-specific innovations such as the Every Newborn Action Plan emphasize scalable health ITs, including machine learning (ML), to reduce neonatal deaths and stillbirths [15]. For example, Rwanda’s intensive care unit network achieved a 30% reduction in neonatal mortality using data-driven protocols [16]. To meet SDG target 3.2, Kenya must leverage technology to accelerate the annual NMR decline from 3.2% to at least 6.5% [17].

#### Role of ML Tools in Reducing Neonatal Mortality in LMICs

ML tools offer potential to enhance triage and clinical decision-making through early prediction, risk stratification, and personalized interventions. ML models developed using multicountry LMIC datasets have demonstrated high accuracy in identifying high-risk neonates, supporting early interventions and improved outcomes [13,18]. ML-enabled mobile health apps such as Ubenwa, which detects neonatal asphyxia from cry analysis, demonstrate practical applicability in resource-limited settings [19]. Integrating ML with health information systems such as DHIS2 further supports real-time, data-driven neonatal care [20].

Despite this promise, ML adoption in LMICs is hindered by digital infrastructure gaps, poor data quality, and limited technical capacity [21]. Effective implementation requires context-specific, user-friendly solutions that align with existing health care workflows. Recognizing this, this study proposes a user-centric evaluation of the neonatal risk predictor variables incorporated into the ML model by Shukla et al [13] (Table 1), assessing their feasibility, applicability, and usability within Kenyan MOH facilities. The study will leverage these predictors from a multicountry LMIC database to determine the model’s potential adoption to reduce neonatal mortality in Kenya.

**Table 1.** Key neonatal variables for predicting perinatal and neonatal risk in low- and middle-income countries (LMICs) according to the machine learning model by Shukla et al [13] (postdelivery scenarios)^a^.

Risk variable	Description and clinical relevance	Contribution to predictive accuracy
Birth weight	Neonatal weight at birth; strongest independent predictor	Highest importance; major impact on model AUC^b^
Bag and mask resuscitation	Need for resuscitation at birth using bag and mask ventilation	Moderate contribution; associated with increased risk
Cluster perinatal mortality	Perinatal mortality rate within the cluster or facility	Contributes to contextual risk stratification
Gestational age at enrollment	Weeks of gestation at the first prenatal visit	Adds modest predictive value
Maternal age	Age of the mother at delivery	Minor contribution to increased risk
Parity	Number of previous births	Minimal change in AUC; included in model
Multiple births	Twin or higher-order deliveries	Minor contribution
Antenatal corticosteroids	Use of steroids before delivery	Slight incremental predictive value
Maternal education: none	Lack of formal maternal education	Weak influence
Hospital delivery	Birth occurring in a hospital setting	Slight predictive value
Maternal antibiotics	Use of antibiotics during pregnancy	Negligible effect
Delivery by physician	Birth attended by a physician	Minor contribution

a The identified postdelivery variables, particularly birth weight and resuscitation requirements, contributed the most to the model’s predictive accuracy, with an area under the curve exceeding 0.80. Prenatal-only variables (ie, maternal age, parity, and antenatal corticosteroids) contributed modestly. The table highlights variables that are considered most actionable for early identification of high-risk neonates in LMIC health facilities.

b AUC: area under the curve.

### Significance of the Study

This study is significant for its potential to reduce neonatal mortality in Kenya, where neonatal deaths account for nearly half of all cases of child mortality under the age of 5 years, highlighting the urgent need for targeted interventions. Despite global and national efforts, progress toward SDG target 3.2, which aims to end preventable newborn deaths and deaths under the age of 5 years by 2030, remains slow, particularly in resource-limited settings. The study evaluates the feasibility, applicability, and usability of neonatal risk predictor variables incorporated into the ML model proposed by Shukla et al [13], which was initially validated in India. By testing these predictors in Kenyan MOH facilities, the study assesses their adaptability to local clinical and infrastructural contexts. Through a user-centered approach, it also explores health care professionals’ experiences, perceptions, and interactions with the tool. If applicable, the model could enable early identification of high-risk neonates, support timely interventions, inform evidence-based policies, promote digital health adoption, and strengthen neonatal care systems, accelerating progress toward SDG 3.2.

### Study Aims and Objectives

#### Overview

This study aims to assess the contextual feasibility, applicability, and usability of neonatal risk predictors incorporated into the ML model proposed by Shukla et al [13] for identifying high-risk neonates in the Kenyan context. The objective is to inform the model’s potential adoption in LMIC settings in alignment with both national priorities and global goals, including SDG target 3.2. The study will evaluate the applicability of these predictors in Kenyan MOH facilities that provide neonatal care and will be conducted between August 2025 and November 2025.

#### Specific Objectives

The specific objectives of the study will be as follows:

- To assess the feasibility of implementing the neonatal risk predictors from the ML model by Shukla et al [13] within triage systems in Kenyan MOH facilities as an LMIC setting
- To determine the applicability of the neonatal risk predictor variables incorporated into the ML model to accurately identify high-risk neonates and their potential contribution to reducing neonatal mortality in line with SDG target 3.2
- To evaluate the usability and user experience (UX) of the ML model’s neonatal risk predictors among health care professionals in identifying high-risk neonates

## Methods

### Overview

A mixed methods approach will be used, combining qualitative and quantitative methods to enhance generalizability and statistical rigor and strengthen the credibility of the findings [22]. The study will be conducted in 3 sequential phases. First, key informant interviews (KIIs) will be conducted to capture contextual insights. The second phase is an intervention period, during which a quantitative neonatal risk survey tool is integrated into clinical workflows to evaluate the contextual feasibility, applicability, and usability of the ML risk predictor variables proposed by Shukla et al [13]. Third, a postimplementation questionnaire will be administered to assess whether the survey tool’s intended outcomes were achieved. Phase 2 (intervention period) and phase 3 (postintervention survey) will follow a longitudinal design, observing the same research participants at 2 time points over a 4-month period to examine changes in outcomes associated with the intervention prior to implementation [23,24]. Therefore, the study will fully leverage the strengths of both qualitative and quantitative methods. These include triangulation, methodological flexibility, in-depth insights, and a comprehensive understanding of the research phenomenon, all of which enhance the validity of the study’s findings compared with a single-method approach [25-27].

### Phase 1: Conducting KIIs

This will be the preintervention phase, which will involve conducting KIIs with key informants (KIs), including facility heads and neonatal department heads at selected MOH facilities providing neonatal care. The KIIs will capture contextual insights into participants’ lived experiences, current workflows, perceptions of neonatal risk interventions, potential benefits, anticipated implementation barriers, and preferred mode of deployment (paper based or electronic). These insights will enable the researchers to better understand the study context and stakeholder expectations, thereby informing the study’s planning and execution.

### Phase 2: Intervention Period

This will be the intervention phase, which will involve providing frontline neonatal care workers at selected MOH facilities with a paper-based neonatal risk assessment tool integrated into routine clinical workflows. Nominal-scale surveys will be used to operationalize and assess the applicability of the risk predictor variables incorporated into the ML model proposed by Shukla et al [13]. The tool will be deployed during routine neonatal care activities over a 4|-month period to enable real-world evaluation of its impact [28]. Longitudinal outcomes will be assessed through observations made during both the intervention and postintervention surveys, focusing on the tool’s feasibility, applicability, usability, and utility. Findings from this phase will inform the potential feasibility of implementing the intervention across Kenyan MOH facilities.

### Phase 3: Postintervention Survey

This will be the postintervention phase, during which quantitative surveys will be conducted with neonatal health workers. The primary objective is to assess whether the intervention’s intended outcomes were achieved, gain a deeper understanding of implementation barriers, highlight areas for improvement, and inform future deployment strategies [29]. Both Likert and semantic differential scales will be used to evaluate the predictors’ usability (ease of use and efficiency), UX (satisfaction and engagement), utility (perceived applicability for identifying high-risk neonates), and feasibility (practicality of implementation) within Kenyan MOH facilities.

Both standardized and custom validation tools will be used to comprehensively assess the usability, UX, interaction satisfaction, and utility of the intervention [30-33]. The instruments will include the Usability Scale Questionnaire, Questionnaire for User Interaction Satisfaction, and a Post-Study Neonatal Risk Predictor Utility Questionnaire. The Usability Scale Questionnaire evaluates UX with the neonatal risk predictor variables using a 5-point Likert scale (1=“strongly disagree,” 2=“disagree,” 3=“neutral,” 4=“agree,” and 5=“strongly agree”). The Questionnaire for User Interaction Satisfaction measures UX and interaction satisfaction using a semantic differential scale with bipolar adjectives such as “terrible”-“wonderful” and “difficult”-“easy.” The Post-Study Neonatal Risk Predictor Utility Questionnaire, a custom questionnaire developed specifically for this study, assesses how well the tool meets user needs and its overall utility. It includes items on ease of use, clarity of instructions, and relevance of content and perceived effectiveness rated on a 7-point Likert scale (1=“strongly agree,” 2=“agree,” 3=“slightly agree,” 4=“neutral,” 5=“slightly disagree,” 6=“disagree,” and 7=“strongly disagree”).

The findings from these tools will provide a comprehensive understanding of the intervention’s applicability and performance and support evidence-based decisions regarding the adoption and implementation of the proposed solution.

### Target Population and Study Setting

The target population will consist of health care workers providing neonatal care services in Kenyan MOH facilities. Specifically, 3 health facilities will be selected from 3 different counties: Kibera Community Health Centre – Amref (Nairobi County), Nazareth Mission Hospital (Kiambu County), and Matuu Level 4 Hospital (Machakos County). To enhance the national representativeness of the study findings, these facilities are located in diverse contexts across Kenya, an LMIC setting, reflecting the unique environmental and socioeconomic conditions in which they operate.

Kibera Community Health Centre is a public, community-based facility located within Kibera, one of Africa’s largest informal settlements characterized by widespread poverty and limited access to basic services, including health care. Nazareth Mission Hospital is a nonprofit, level 5 tertiary referral hospital in Kiambu County. It serves patients from across Kenya and neighboring regions, offering a wide range of specialized services. Matuu Level 4 Hospital, located in Machakos County, is a public level 4 health facility operating in a semiarid region characterized by low-income households, extreme temperatures, water scarcity, and food insecurity.

### Sampling Procedure

#### Overview

Textbox 1 summarizes the inclusion and exclusion criteria, detailing the specific conditions that determine participant eligibility for inclusion in or exclusion from the study.

Textbox 1. **Inclusion and exclusion criteria for study participants.**

**Inclusion criteria**

- Aged 18 years or older
- Health care professionals (eg, clinicians, nurses, midwives, and pediatricians) directly involved in neonatal care
- Current assignment to neonatal care duties at one of the selected study facilities during the study period
- At least 6 months of continuous experience providing neonatal care
- Willingness and ability to provide informed consent

**Exclusion criteria**

- Temporarily rotating through neonatal units for training, internship, or short-term placement
- Exclusive assignment to nonneonatal departments (eg, outpatient, maternity only, or administrative roles) during the study period
- On extended leave or otherwise unavailable during the intervention period
- Prior direct involvement in the design or development of the neonatal risk assessment tool
- Less than 6 months of continuous experience providing neonatal care

#### Qualitative Sample

Participants will include KIs from the selected health facilities. These will primarily include senior staff (ie, heads of neonatal units or senior clinicians involved in neonatal care). Owing to their administrative roles and clinical experience, these individuals are well positioned to provide informed insights into the study context, including the perceived value, potential impact, and implementation barriers of the neonatal risk screening tool [34]. Owing to the limited number of eligible KIs per facility, a purposive sampling approach will be used. While qualitative sample sizes are commonly determined by data saturation [35,36], a minimum of 3 KIIs will be conducted, one in each study site (ie, Kibera, Nazareth, and Matuu). Restricting participation to 1 KI per facility is considered appropriate as qualitative findings will be supplemented with quantitative data.

#### Quantitative Sample

Potential participants will primarily comprise health care workers involved in providing neonatal care at the selected facilities, including neonatal pediatricians, clinicians, general practitioners, nurses, and midwives. Across the 3 study sites, 43 staff members provide neonatal care, as shown in Table 2. However, due to staffing constraints, the same personnel often rotate across multiple departments, including neonatal, maternity, and postnatal units, and therefore are not permanently stationed within neonatal units.

**Table 2.** Number of neonatal health care workers at the selected facilities^a^.

Facility name	Qualitative sample (senior health care workers; n=3), n (%)	Quantitative sample (frontline health care workers), n (%) $n=\frac{N}{1+N(e^{2})}$	
		Frontline care workers (n=43)	Required sample size, n (%)
Kibera Community Health Centre	1 (33.3)	11 (25.6)	11 (26.8)
Nazareth Mission Hospital	1 (33.3)	17 (39.5)	16 (39.0)
Matuu Level 4 Hospital	1 (33.3)	15 (34.9)	14 (34.1)

a The qualitative sample comprises purposively selected senior neonatal health care workers in leadership or supervisory roles. The quantitative sample includes frontline health care workers directly providing neonatal care at each facility during the study period. Sample totals reflect eligible and available participants at the time of data collection. Frontline health workers will collect data on the specified predictive variables from 400 newborns across the 3 selected facilities, distributed as follows: 100 in Kibera Community Health Centre, 100 in Nazareth Mission Hospital, and 200 in Matuu Level 4 Hospital.

Given the study’s focus on assessing the applicability of neonatal risk parameters in routine clinical settings, only staff assigned to neonatal care units during the study period will be eligible to participate. On the basis of this criterion, a purposive sample of 10 health care workers will be recruited across the 3 sites: Kibera (n=4, 40%), Matuu (n=4, 40%), and Nazareth (n=2, 20%). This approach ensures the inclusion of staff actively providing neonatal care during the study period by integrating the proposed neonatal risk parameters into routine clinical workflows [37].

Furthermore, according to the literature, total population sampling is appropriate when the population with the specific characteristics of interest is very small [38-41], which is the case here. The researchers believe that neonatal care workers in the selected facilities are uniquely equipped with the knowledge, skills, experience, and exposure necessary to provide meaningful responses that can inform the study. The total number of frontline neonatal care workers across the 3 facilities is 43, as shown in Table 2. Applying the Yamane [42] formula to calculate the required quantitative sample size, the study anticipates recruiting 11 participants from Kibera, 14 from Nazareth, and 16 from Matuu, yielding a total sample of 41 participants (Table 2).

### Data Collection and Analysis 

Data will be collected using both qualitative and quantitative methods, including KIIs, neonatal risk assessment tool and questionnaire, and postintervention questionnaires.

### Data Capture and Management Plan

#### Overview

Data will be entered into a secure electronic database by trained personnel and subjected to routine verification and quality checks. Access to the data will be restricted to authorized study staff to ensure confidentiality, privacy, and data integrity. All data handling procedures will comply with Kenyan data protection laws governing the privacy and confidentiality of personal information. Paper-based records will be securely stored in locked cabinets at each facility, whereas electronic data will be encrypted and password protected. Study data will be used solely for research purposes and will not be accessible to individuals or entities outside the project team.

#### Qualitative Data Collection

KIIs will be conducted with KIs at the selected facilities in 2 stages. The initial KIIs will be carried out to collect baseline data aimed at understanding current workflows, existing challenges, and expectations related to the use of the neonatal risk predictors proposed in the ML model by Shukla et al [13]. Follow-up KIIs will also be conducted at the end of the intervention period to explore participants’ lived experiences, perceived benefits, challenges encountered, and suggestions for improving the effectiveness of the predictors.

#### Quantitative Data Collection

The study will recruit 1 co–principal investigator from among neonatal care clinicians or staff at each selected facility. The co–principal investigators will be responsible for administering a paper-based survey tool (questionnaire) containing the neonatal risk predictors incorporated in the study by Shukla et al [13] on intrapartum stillbirth and neonatal mortality. The tool will be used by fellow staff members as part of routine neonatal care operations during the intervention period. These risk factors are considered significant neonatal predictors and are assessed at 2 time points: delivery (day 1) and after delivery (day 2). They are grouped into 5 key categories, namely, maternal, pregnancy, delivery, neonatal, and after delivery.

It is essential to note that the data collected during the study will not be used to input into the specific predictive ML model to generate actual risk scores. Instead, the study will focus on gathering input data to assess the feasibility, usability, and applicability of the risk predictors within the Kenyan context prior to full implementation of the ML prediction tool. After the intervention period, participants will be administered postsurvey questionnaires comprising closed-ended questions and several measurement scales. These surveys will collect feedback on the feasibility, usability, UX, and perceived utility of the neonatal risk predictors to inform future decisions regarding the tool’s adoption.

### Data Analysis

Qualitative data will be analyzed using thematic analysis to identify patterns and themes emerging from the KIIs. The analysis will follow an inductive coding approach involving systematic identification and classification of concepts, categories, and subcategories to generate overarching themes grounded in the data [5,43,44]. Data analysis will be conducted using the NVivo qualitative data analysis software (Lumivero). Thematic analysis is particularly valued for its flexibility and accessibility, making it a versatile qualitative research method. It enables in-depth exploration of complex datasets and supports the generation of rich, nuanced insights while remaining relatively straightforward to learn and implement [45].

Quantitative data obtained from the surveys will be analyzed using descriptive statistical techniques, including calculating frequencies and percentages and presenting results in tables and charts. Descriptive statistics are particularly useful for summarizing complex numerical data into manageable and interpretable formats, thereby facilitating the identification of key patterns and trends [46]. Data analysis will be conducted using Stata (StataCorp). This approach enhances clarity, improves communication of findings, and supports timely, evidence-based decision-making.

### Ethical Considerations

Ethics approval (SU-ISERC2949/25) was received from the Strathmore University Institutional Scientific and Ethical Review Committee in Nairobi, Kenya, on July 28, 2025, prior to this study. Various ethical considerations related to key research stakeholders will be carefully addressed, including concerns involving the researcher, research participants, and sponsoring organization [47]. The study will strictly adhere to these ethical guidelines to ensure compliance and integrity throughout the research process. Informed consent was obtained before engaging participants, after explaining the aim, benefits, and risks associated with the study and the mechanisms put in place to address them. In addition, privacy and confidentiality were ensured by omitting identifying details.

## Results

### Status

The study was funded in March 2025. Six participants (frontline neonatal health workers) had been recruited by the time of initial submission of the manuscript (August 2025). Data analysis is ongoing and is expected to be concluded in March 2026. Publication of study findings is expected by June 2026.

### Feasibility of Implementation

The research will provide a clear understanding of whether and how the neonatal risk predictors developed by Shukla et al [13] will be practically integrated into existing triage systems within Kenyan health care facilities. This includes identifying both enablers of and barriers to implementation in LMIC settings, assessing health system readiness, the availability of resources such as skilled personnel and infrastructure, and the level of institutional support for adoption.

### Applicability and Clinical Impact

The study will explore the applicability and clinical impact of the tool in accurately identifying high-risk neonates. It will investigate whether early risk identification using the tool improves neonatal outcomes or enables more timely interventions. Ultimately, the research will assess the tool’s potential to reduce neonatal mortality, aligning with SDG target 3.2, which aims to end preventable deaths of newborns.

### Usability and UX

The study will assess the usability and UX of the ML model’s neonatal risk predictors by examining how health care professionals interact with and perceive the usefulness of these variables in real-world clinical settings. This assessment will evaluate the predictors’ learnability and efficiency and overall user satisfaction, as well as the tool’s seamless integration into routine clinical workflows. Additionally, user feedback will provide valuable insights into the relevance, clarity, and acceptability of the predictors, helping inform potential improvements and ensure that they meet the practical needs of their intended users.

### Research Impact, Use, and Dissemination of Results

Evaluating the utility of the ML tool’s predictors is crucial for understanding their applicability in identifying neonatal risks. This evaluation will inform strategies for potential implementation and support the tool’s sustained use, maximizing its impact and application in practice. The tool is expected to have a positive impact on neonatal care processes across health facilities in Kenya. Anticipated benefits include improved triage decisions, enhanced applicability in neonatal care delivery, increased operational efficiency, and greater productivity among health care providers.

Dissemination of the study findings will occur at 2 levels. First, preliminary results will be presented to the stakeholders at a local conference. Second, the results will be shared through 2 peer-reviewed scientific journal publications. Throughout the dissemination process, the project will adhere to key principles aligned with responsible publication practices. These include maintaining ethical research conduct throughout the study, respecting intellectual property rights by properly acknowledging original authors and their work, and upholding academic integrity. The latter involves conducting the publication process with honesty, fairness, transparency, respect, and accountability, ensuring that the research makes a credible contribution to the broader scientific and health care communities.

## Discussion

### Expected Findings

Neonatal health outcomes remain a significant public health concern in LMICs, where approximately 98% of global neonatal deaths occur due to preventable causes such as preterm birth, intrapartum complications, and infections [48]. SSA bears a disproportionate burden, with NMRs often exceeding 25 per 1000 live births [49]. Socioeconomic disparities are key contributors as infants born to mothers with low literacy and lower income levels are more vulnerable to poor health outcomes. Access to prenatal care and essential neonatal services is often hindered by financial constraints, transport challenges, and low health literacy [50].

Moreover, fragile health care systems in LMICs lack adequate infrastructure, including electricity, medical equipment, and essential medical commodities and supplies, which compromises the quality of neonatal care. Inadequate infection control further exacerbates neonatal risk, with sepsis remaining a leading cause of mortality. Despite these challenges, promising interventions have emerged, such as community health worker–led programs and decentralized neonatal care units, which have shown substantial reductions in mortality. Achieving SDG 3.2 will require integrated efforts focused on health system strengthening; equitable access to care; and targeted community-based strategies, including digital health interventions such as ML tools.

The impact of contextually informed usability studies in LMICs extends beyond tool optimization to strengthening health system resilience. Effective usability testing has enabled the scalable deployment of artificial intelligence–driven diagnostic aids in Uganda, where simplified interfaces have reduced clinician cognitive load and improved diagnostic accuracy by 25% [51,52]. Moreover, integrating usability feedback into national digital health policies, as piloted in Rwanda’s health information exchange, fosters sustainable innovation [53]. Future efforts must prioritize capacity building for local usability researchers and leverage mixed methods data by combining quantitative metrics (such as task success rates) with qualitative insights to advocate for resource allocation. Ultimately, embedding usability science in LMIC health initiatives bridges this significant gap in technology adoption, advancing equity in global health delivery.

Several well-established frameworks guide the measurement of the feasibility of health care interventions such as digital health tools. Notably, the reach, effectiveness, adoption, implementation, and maintenance framework comprehensively addresses feasibility by evaluating the real-world effectiveness of interventions [54]. Additionally, the Medical Research Council framework for complex interventions provides guidance on the iterative assessment of interventions’ practicality, effectiveness, and sustainability in diverse contexts [55]. Other frameworks include implementation mapping, which systematically connects implementation determinants with specific strategies, providing a structured approach to assessing feasibility and developing tailored solutions [56,57]. The Consolidated Framework for Implementation Research also provides a detailed framework for analyzing feasibility, considering elements such as intervention characteristics, inner and outer settings, and stakeholder engagement, often in conjunction with pragmatic tools such as the Pragmatic Context Assessment Tool and the Clinical Sustainability Assessment Tool [58].

Successful adoption of ML tools within health care systems is influenced by stakeholder engagement and organizational readiness. The literature highlights the critical role of clinician and organizational perceptions in determining the feasibility, acceptability, and appropriateness of new technologies [59-61]. Complex systems thinking frameworks emphasize that feasibility assessments must account for dynamic interactions among technology, workflows, and human factors within health care environments [47,62,63]. Research indicates that early and sustained stakeholder engagement across clinical, administrative, and IT teams, as well as end user populations such as patients and caregivers, significantly enhances the feasibility of adopting ML tools. Additionally, assessing organizational readiness by evaluating knowledge, attitudes, and contextual factors (such as existing infrastructure and resource constraints) ensures that ML tools are tailored to local needs, thereby enhancing their practical integration and sustained use within health care systems [64-66].

The proposed evaluation of the ML model by Shukla et al [13] for neonatal risk prediction in Kenya is vital in informing its adoption and alignment with SDG target 3.2. It must address local challenges such as delayed triage, limited resources, inadequate staff training, and weak digital infrastructure. Mobile health platforms, already used in Kenyan CHW programs, present a scalable solution [67,68]. Integrating the tool with Kenya’s electronic medical record systems could enable real-time alerts for high-risk neonates. A community validation in Kakamega County found that CHWs achieved 85% accuracy with digital triage tools after brief training [69]. Evaluating usability in Kenya may improve triage, referrals, and resource use, thus reducing neonatal mortality. This initiative can guide scale-up, infrastructure investment, and policy alignment, supporting digital health transformation in LMICs.

### Strengths and Limitations of the Study

The project will adhere to established quality standards throughout its life cycle, encompassing planning, data collection and analysis, and reporting of findings. Quality assurance in research refers to the techniques and strategies used to ensure proper care and control during the research process [70]. To uphold quality assurance in research, the project will implement measures to enhance research credibility, accuracy, and transparency [71,72]. First, staff competence will be ensured by providing relevant training to equip personnel with the necessary skills to perform their roles effectively. Project planning and documentation will be transparent, with all essential information made accessible and understandable to stakeholders, promoting open communication. Standard operating procedures will be established to guide project execution, including clear objectives, prioritized activities, appropriate methodologies, clear communication, and the protection of participant and data confidentiality.

However, this study has several limitations inherent to its exploratory preimplementation design. Neonatal health care staff will collect data by integrating a paper-based checklist into routine workflows, which may introduce observer and Hawthorne effects. The absence of a control group and blinding due to limited staffing and purposive recruitment of active neonatal care providers restricts causal inference and may introduce bias, although participants’ prior experience allows for meaningful before-and-after comparisons. To mitigate these limitations, methodological triangulation using both qualitative and quantitative approaches [73], including KIIs, surveys, and expert reviews, will be applied, alongside defined data quality standards. Data will be securely managed through restricted access, routine verification, encryption, and locked storage in compliance with Kenyan data protection laws. Ethical oversight, participant validation, expert review, and peer-reviewed publication will further strengthen the study’s credibility and trustworthiness.

## Abbreviations

KI: key informant
KII: key informant interview
LMIC: low- or middle-income country
ML: machine learning
MOH: Ministry of Health
NMR: neonatal mortality rate
SDG: Sustainable Development Goal
SSA: sub-Saharan Africa
UX: user experience
